# Antibacterial activity of *Staphylococcus aureus* biofilm under combined exposure of glutaraldehyde, near-infrared light, and 405-nm laser

**DOI:** 10.1371/journal.pone.0202821

**Published:** 2018-08-27

**Authors:** Van Nam Tran, Chakradhar Dasagrandhi, Van Gia Truong, Young-Mog Kim, Hyun Wook Kang

**Affiliations:** 1 Department of Biomedical Engineering, Pukyong National University, Busan, Korea; 2 Marine-Integrated Bionics Research Center, Pukyong National University, Busan, Korea; 3 Department of Food Science and Technology, Pukyong National University, Busan, Korea; 4 Center for Marine-Integrated Biomedical Technology, Pukyong National University, Busan, Korea; Massachusetts General Hospital, UNITED STATES

## Abstract

Healthcare-associated infections have increasingly become problematic in the endoscopic procedures resulting in several severe diseases such as carbapenem-resistant Enterobacteriaceae (CRE)-related infections, pneumonia, and bacteremia. Especially, some bacterial strains are resistant to traditional antimicrobials. Therefore, the necessity of developing new antibiotics or management to deal with bacterial infections has been increasing. The current study combined a low concentration of glutaraldehyde (GTA) with near-infrared (NIR) light and 405-nm laser to entail antibacterial activity on *Staphylococcus aureus* biofilm. MTT (3-(4,5-dimethylthiazol-2-yl)-2,5-diphenyltetrazolium bromide) assay and colony forming unit (CFU) counting were used to quantify the viable cells while fluorescent and scanning electron microscopic images were used to qualitatively evaluate the cell membrane integrity and structural deformation, respectively. Practically, *S*. *aureus* biofilm was highly susceptible (7% cell viability and 6.8-log CFU/cm^2^ bacterial reduction for MTT assay and CFU analysis, respectively) to the combination of GTA (0.1%), NIR light (270 J/cm^2^), and 405-nm laser (288 J/cm^2^) exposure. GTA could form either DNA-protein or protein-protein crosslinks to inhibit DNA and protein synthesis. The NIR light induced the thermal damage on protein/enzymes while 405-nm laser could induce reactive oxygen species (ROS) to damage the bacterial membrane. Thus, the proposed technique may be a feasible modality for endoscope cleaning to prevent any secondary infection in the healthcare industry.

## Introduction

A biofilm is a collection of microorganisms that irreversibly adhere to many various surfaces of materials or tissues [1, 2]. These microorganisms are normally embedded in a self-produced matrix of extracellular polymeric substances (EPS), which mainly consist of extracellular bacterial DNA, proteins, exopolysaccharides, and enzymes [3–5]. EPSs play a vital role as a “bacterial protector” against any antibiotic penetration and the cellular strike by host innate immune cells [3]. As a result, the bacterial biofilm is responsible for many common sources of intractable diseases such as CRE-related infections, pneumonia, and bacteremia [1, 2, 4, 6–9].

Many scientists claimed that the second highest percentage of human death in all over the world has originated from pathogenic bacterial infections, accounting for around 17 million people per year worldwide [3, 10, 11]. Undesirably, some of the pathogens have adapted and resisted to conventional drugs and antibiotics. In recent years, antibiotic impermeability has been increasingly observed in a plenty of multidrug-resistant bacteria, for instance, methicillin-resistant *S*. *aureus* [12]. In fact, the factors resulting in bacterial resistance in biofilm can be divided in three groups: biomedical factors (exopolysaccharides, antibiotic-degrading enzymes, extracellular DNA, efflux pumps, and quorum sensing), molecular mechanism (lateral/horizontal gene transfer, and mutation), and altered host factors (oxidative stress, chemical signals, toxin-antitoxin modules, temperature, pH, and cell density) [13]. Therefore, the development of new antibiotics and management for dealing with bacterial infections has been urgently considered to prevent any chronic and obstinate diseases from happening.

In order to tackle serious health-associated infections (HAIs) due to biofilm, many types of antimicrobial research have been conducted in terms of hospital disinfection and endoscope reprocessing [14–16]. The current methods for hospital disinfection include chemotherapy (i.e., using antibiotics [17, 18], antibacterial peptides [19], peptide-polysaccharides [20], cationic materials [10], antibacterial coatings [21, 22], and polymers [23, 24], photo-thermal therapy (PTT) [25–30], and photodynamic therapy (PDT) [31–33]. Meanwhile, in the field of endoscope reprocessing, some standard disinfectant solutions such as 2% GTA, 0.55% orthophthalaldehyde (OPA), and 0.15% peracetic acid (PAA) solutions have been widely used in the clinical applications [34–36]. Among others, 2% GTA offers more advantages to endoscope reprocessing such as excellent biocidal activity, relative inexpensiveness, no degradation to endoscopes, and no corrosion to metals, rubbers and plastics [37–39].

On the other hand, PTT can disrupt the biofilm structure based upon the physical heat that is generated by light absorption of materials or tissues [27, 29]. In practice, NIR light (wavelength = 0.7 ~ 1.1 μm) demonstrates tremendous benefits in terms of inactivating bacterial biofilm due to the outstanding capability of tissue penetration and minimal injury to the healthy cells [3, 25]. In fact, the NIR-based PTT approach to kill bacteria has widely been applied for the last few years. For instance, Wu *et al*. synthesized a graphene-based photo-thermal agent (magnetic reduced graphene oxide functionalized with GTA—MRGOGA) for efficiently and effectively sterilizing *S*. *aureus* upon NIR laser exposure [25]. Many other scientists have applied nanoparticle-based high absorption materials for removal of the biofilm in order to improve the thermal effects due to their great stability, controllable size, and good biocompatibility [26–30].

Similarly, PDT using 405-nm laser for the bacterial inactivation has been elucidated in plenty of previous studies [12, 40, 41]. Maclean *et al*. agreed that 405-nm wavelength could induce the peak germicidal activity via photoexcitation of porphyrins and oxidative damage to the bacteria [12]. In fact, photoactive compounds including porphyrins can decontaminate bacteria by producing ROS due to the absorption of 405-nm light. As being cytotoxic to the bacterial cells, the generation of ROS results in damage to DNA/protein/ lipid and eventually cell death [41].

Although 2% GTA disinfectant brings many benefits into clinics (bacterial fixation and disinfection), the main limitations include serious irritation to human respiratory tract and skin, protein coagulation, and cost due to no reusability [38, 39]. By contrast, in order to sterilize the target, PTT using NIR light induces high temperature on the biofilm surface, which can, as a result, damage rubber and plastic surficial materials [9, 42]. On the other hand, PDT using 405-nm laser shows less efficacy of killing certain bacteria than UV-C (wavelength = 260 ~ 270 nm) and UV-B light (wavelength = 280 ~ 400 nm), especially in liquid suspensions, compared with exposed surfaces [12, 43]. Thus, the current study combined a low concentration of GTA solution with low doses of NIR light and 405-nm laser to entail antibacterial activity on *S*. *aureus* biofilm.

## Materials and methods

### Photo and chemical agent preparation

In the current study, a therapeutic near-infrared bulb (250W, spectral range = 780 nm ~ 1060 nm, working diameter = 176 mm, HH-2500, Philips Inc., South Korea) was used to kill bacteria by the heat due to low cost and excellent capability of tissue penetration in earlier reports [3, 27]. The spatial light distribution of the bulb was visually measured by using a digital camera (Canon-70D-18-55, Canon Park, Melville, New York). The measurement distance from the output surface of the therapeutic bulb to the screen (background) was set to be identical in the treatment (distance = 20 cm). To deliver the 405-nm laser source, a customized flat fiber was prepared in the following manner. A multimode fiber with 400-μm core diameter (FIP400440480; Molex Inc., Wellington Court, Lisle, IL, USA) was firstly cut in a segment of 2 m, the two fiber tips were then stripped out about 3 mm and cleaned by 99.7%-acetone liquid (SamChun Pure Chemical Co., Ltd, Gyeonggi-do, Korea). Once all cladding and buffer layers were removed, both fiber tips were flatted out by using the grinding machine (the Radian^TM^ Polisher, Krelltech Inc., Neptune City, NJ). Then, one fiber tip was linked with a SMA-905 connector (Thorlabs Inc., Newton, New Jersey) and the other was covered by using a quartz-glass cap to protect any mechanical injury during the usage. To evaluate the light emissions from the flat fiber, HeNe light accompanied in the 405-nm laser system (also called as a laser navigator) was used in both photographed and goniometric manners. For goniometric measurement, the flat fiber was azimuthally mounted around the detector in the customized goniometer (rotational radius = 4 cm). The measurement speed was set to be 0.03 rad/s. For the chemical preparation, GTA, propidium iodide (PI), ethidium bromide (EtBr), and alcohol were purchased from Sigma Aldrich (St Louis, USA). All other chemicals and reagents are of analytical grade.

### Cultures and growth conditions

*Staphylococcus aureus* ATCC 6538 was purchased from American Type Culture Collection (USA). The strain was maintained on glycerol stocks at -80°C. Overnight grown cultures in tryptic soy broth were used for biofilm experiments. Before experiments, the strain was first activated in 37°C in phosphate-buffered saline (PBS) followed by inoculating on Trypticase Soy Agar (TSA) (Sigma-Aldrich Co., St. Louis, MO, USA) for 24 hours. A colony was transferred into tryptic soy broth (TSB) (Difco, BD Sciences, USA) and grown overnight at 37°C with shaking (120 rpm) in an orbital shaker (labTech, Daihan Labtech Co., Ltd, Korea). Then the culture was diluted 100 fold in tryptic soy broth (TSB) to obtain initial inoculums of 5×10^5^ colony forming units (CFU) per ml.

### Establishment of staphylococcal mature biofilms

Mature biofilms of *S*. *aureus* ATCC 6538 was established according to the method previously described [44]. Briefly, the overnight grown culture of *S*. *aureus* ATCC 6538 adjusted to 5x10^5^ CFU/ml) was inoculated into 24 well polystyrene tissue culture plate containing 1 ml TSB (+ 1% glucose) and incubated stationary at 37°C for 24 h. At the end of the incubation, the biofilms in the tissue culture plates were gently rinsed with phosphate buffer saline (PBS, pH = 7.4) twice, and the biofilms were gently air dried for 5 min. Later, the biofilms in the plates were subjected to treatment (GTA, NIR, and 405-nm laser) as described in the experimental design section below.

### Experimental setup

**Fig 1:**
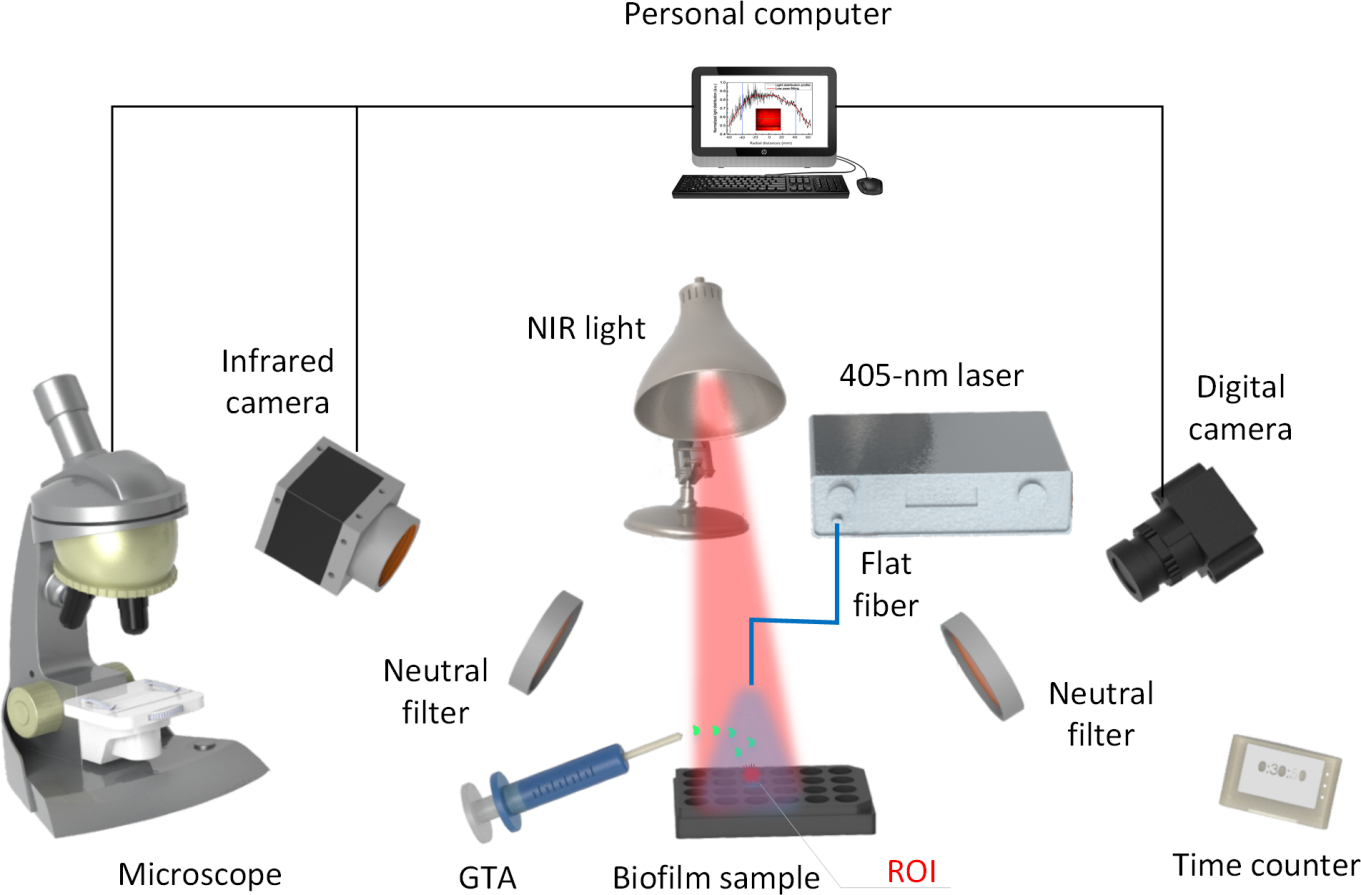
Experimental set-up. Antibacterial activity of *S*. *aureus* biofilm under combined exposure of GTA, NIR light, and 405-nm laser (ROI: region of interest).

Fig 1 illustrates a conceptual experimental set-up for the current *in vitro* study. Before treatment, all samples were quickly checked by using a stereo microscope (HSZ, Huvitz Co., Gyeonggi-do, South Korea) to evaluate whether the biofilm was uniformly formed on the well plate surface. For the chemical disinfection process, GTA solution (concentration = 25%) was serially diluted two folds using PBS (pH = 7.4). The samples were then exposed to the NIR light (average irradiance = 1.5 W/cm^2^) to further dehydrate the biofilm surface with moderate temperature and treated by using 405-nm laser delivered through the flat fiber. The fiber was positioned around 4 cm above the biofilm surface (beam spot diameter = 18 mm). Therefore, the laser beam spot covered the entire area of the treated well (well diameter = 15.6 mm, well area = 1.91 cm^2^). To monitor the spatial-temporal temperature development during the treatment, an infrared camera (FLIR A325, 320 × 240 pixels, resolution = 25 μm, spectral range = 7.5 ~ 13 μm, FLIR, Wilsonville, Oregon) was deployed at around 50 cm above the biofilm surface.

To investigate the optimal therapeutic conditions of all germicide, the biofilm samples were sequentially tested by altering various variables (germicidal type, light irradiance, and exposure time). It should be noted that fluence (J/cm^2^) is mathematically the production between the irradiance (W/cm^2^) and the exposure time (s). Initially, six concentrations of GTA solution (0.05%, 0.1%, 0.25%, 0.5%, 1%, and 2%) were selected for the alone testing in 180 s based upon the standard reprocessing of endoscope (2%) [37, 39]. Similarly, alone NIR light was also attempted in three conditions (90, 270, and 450 J/cm^2^). On the other hand, 405-nm laser alone was tested either by maintaining the irradiation time (60 s) and changing the irradiance (0.4, 0.8, 1.2, and 1.6 W/cm^2^) or maintaining the irradiance (1.6 W/cm^2^) and changing the irradiation time (60, 180, and 300 s). Furthermore, in order to explore the combined effects of NIR light and 405-nm laser, five groups (one for control) were sampled to test for 60-s NIR irradiation (fluence = 90 J/cm^2^) along with various 405-nm irradiance (0.4, 0.8, 1.2, and 1.6 W/cm^2^) for 60 seconds. The experiment was then repeated with the higher energy of NIR exposure (fluence = 450 J/cm^2^) to inspect further biofilm damage. To completely confirm the bacterial killing effects of NIR light and 405-nm laser, the extreme condition of NIR light (fluence = 450 J/cm^2^) in conjunction with 405-nm laser (fluence = 480 J/cm^2^) was studied. Eventually, GTA (concentration = 1%), NIR light (fluence = 270 J/cm^2^), and 405-nm laser (fluence = 288 J/cm^2^) were proposed as an effective combination to disinfect biofilms of *S*. *aureus* formed on polystyrene plates.

#### Quantitative assessment of biofilms

Anti-biofilm effect of individual germicide agents (GTA, NIR light, and 405-nm laser) or its combinations thereof was determined by using MTT assay and bacterial viable count determination. The dosage regiment of chemical and physical germicide agent is briefly described in S1 and S2 Tables (Supporting information).

#### Evaluation of biofilm viability by MTT assay

Biofilms were firstly washed with PBS (pH = 7.4) and suspended in PBS (1 ml). The suspensions were then treated with 50 μl MTT solution (Stock, 2 mg/ ml) and incubated at 35°C for 3 h. The MTT formazan formed was extracted with dimethyl sulfoxide (DMSO) and the absorbance of the purple formazan product was determined at 575 nm using a microplate reader (Multiskan GO, Thermo Fisher Scientific Korea Ltd., Seoul, Korea).

#### Cell viability determination by CFU analysis

*S*. *aureus* ATCC 6538 biofilms were grown for 24 h on 24-well polystyrene plates and exposed either to GTA, NIR light, and 405-nm laser or their combinations. The treated biofilms in the polystyrene plates were detached by using sterile tweezers and suspended in 1 ml PBS (pH = 7.4). The aggregated cells were dislodged by brief vortexing, and the resulting culture suspension (1 ml) was added to 9 ml of sterile PBS (pH = 7.4). Then, 1 ml aliquot was serially diluted 10 folds. A 0.1-ml aliquot from each dilution was aseptically withdrawn, spread on the TSA plates, and incubated at 35°C for 24 h before viable counts were determined.

### Membrane integrity measurement

Membrane integrity of *S*. *aureus* ATCC 6538 biofilms in presence of germicide treatment was determined by using confocal laser scanning microscopy (CLSM). Briefly, the mature biofilms (5 × 10^8^ CFU/ml) formed on polystyrene plates were treated either with GTA (0.1%), NIR light (270 J/cm^2^), and 405-nm laser (288 J/cm^2^) alone or their combinations. The polystyrene plates were washed twice with PBS (pH = 7.4) to remove the non-specific binding cells, and the hard biofilms were incubated with PI (5 μM) in 1 ml PBS for 5 min. The biofilms were rinsed with PBS and subsequently incubated with counterstain, DAPI (5 μM) in 1 ml PBS for 30 min. The biofilm cells upon washing with PBS were observed under confocal laser scanning microscopy (CKX53, Olympus Inc., Tokyo, Japan) equipped with 40x objective lenses, using the red channel (λ_ex_ 504, λ_em_ 540) and the blue channel (λ_ex_358, λ_em_ 461). For each treatment, images were taken at random locations (N = 5), and representative images were presented.

### Scanning electron microscopy

Structural changes induced by the germicide treatment (GTA, NIR light, and 405-nm laser) at the optimal concentrations against mature biofilms of *S*. *aureus* ATCC 6538 were determined by SEM studies. The biofilm was grown on polystyrene chips (1 × 1 cm^2^) in 24-well plates for 24h and were treated with germicides (GTA, NIR light, and 405-nm laser, or their combinations) for the indicated time. Then, the samples were washed twice with PBS (pH = 7.4) and fixed by using 2.5% GTA in 0.1 M PBS (pH = 7.2) for four hours at 4°C. The fixed samples were then dehydrated in the increasing concentrations of ethanol (10, 25, 50, 75, and 100%) and isoamyl alcohol (100%) for 10 min. The polystyrene chips were finally air dried, mounted and then sputter coated with gold-palladium and analyzed under the scanning electron microscope (S-3400N, Hitachi, Tokyo, Japan). Bacteria treated with PBS (pH = 7.4) without the addition of germicides processed in the same manner was used as the controls.

### Statistical analysis

In this study, data was presented as the mean ± standard deviation (SD). Nonparametric statistical differences between the treated and untreated groups (controls) were tested for significance by using Mann-Whitney U test. The calculations were conducted by using SPSS program (SPSS Inc., Chicago, Illinois), and *p* < 0.05 was regarded as significant.

## Results

### Light distribution measurement

The normalized spatial light distribution from the therapeutic NIR bulb (S1A and S1B Fig) and 405-nm laser system (S1C and S1D Fig) was presented in 2-D and 3-D perspectives (Supporting information). It was noted that the green and yellow lines depict how both beam profile graphs were analyzed. Overall, the NIR bulb uniformly irradiated the light over the wide area (radius = 40 mm) although the peak intensity was 13% higher than that at 40-mm boundary. Therefore, the infrared beam spot was large enough to irradiate a half of 24-well plates (80 mm × 80 mm), in which the biofilm was cultured. S1B Fig reflected 3-D light emission mapping from the digital image in S1A Fig. Similarly, the light distribution was almost uniform in the 80-mm diameter region. The same reflection between 2-D and 3-D light distributions was compared for the 405-nm laser. The blue line in S1C Fig shows the normal Gaussian distribution with the standard deviation of 9 mm (beam spot size = 18 mm) measured by the customized goniometer while the red line represents its Gaussian fitting line. Moreover, the beam profile was visually confirmed by 3-D mapping in S1D Fig to show no difference between the two perspectives.

### Effect of germicide alone on biofilm viability

**Fig 2:**
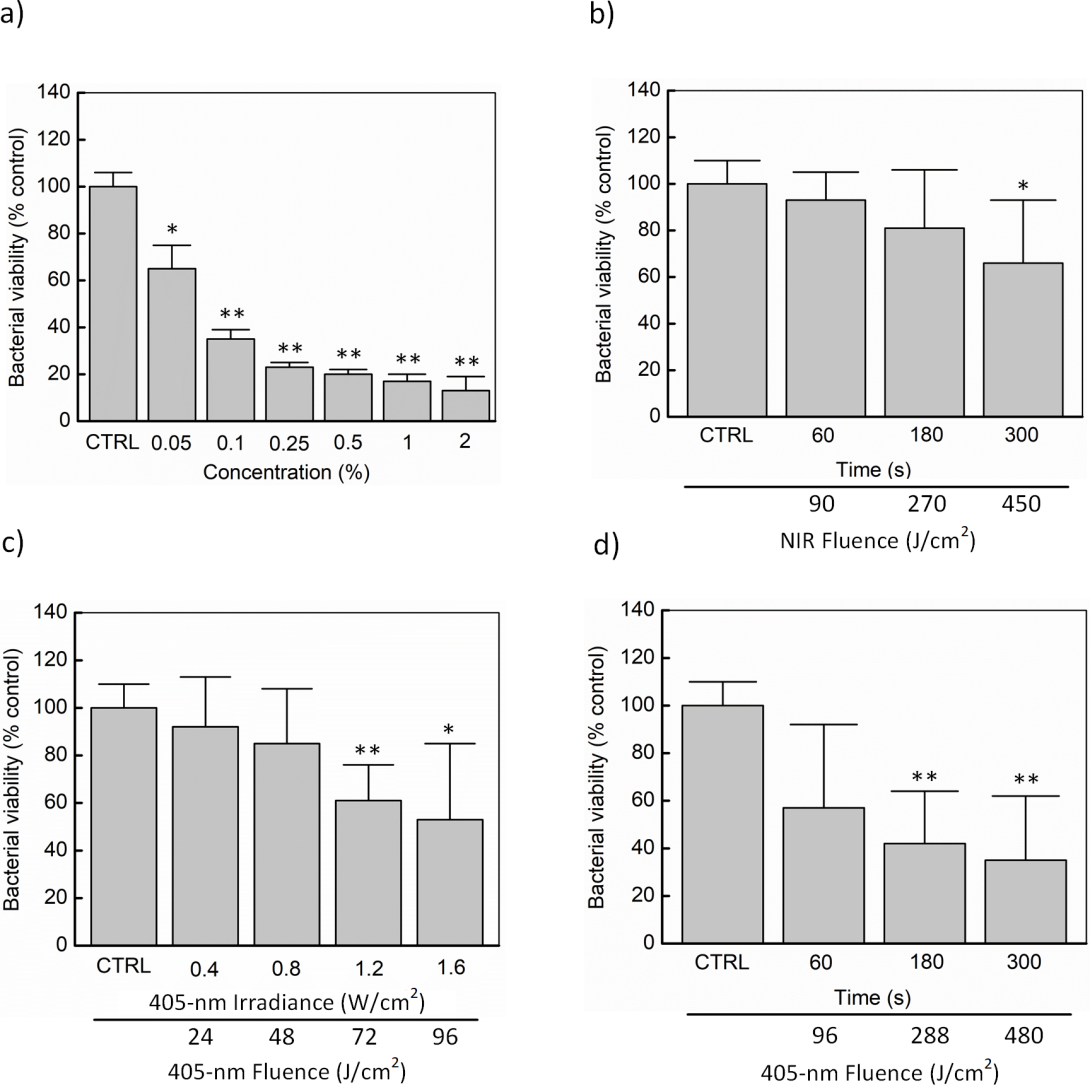
Biofilm decontamination on polystyrene surface (24-well plate surface) by each germicide. (a) GTA exposure at various concentrations of 0.05, 0.1, 0.25, 0.5, 1, and 2%. (b) NIR light exposure for 60, 180, and 300 s (irradiance = 1.5 W/cm^2^). (c) 60-s 405-nm laser exposure at various irradiances of 0.4, 0.8, 1.2, and 1.6 W/cm^2^. (d) 1.6-W/cm^2^ 405-nm laser exposure for 60, 180, and 300 s. The biofilm quantity was estimated by measuring the metabolic activity of the biofilms with MTT assay (*p < 0.05 and **p < 0.01 vs. untreated control (CTRL); N = 5).

Fig 2 exhibits the results of biofilm decontamination on a polystyrene surface (24-well plate surface) by each germicide (i.e., GTA, NIR light, and 405-nm laser; N = 5). Overall, the inactivation kinetics of *S*. *aureus* was presented as a function of dose. Firstly, Fig 2A illustrates the dose-dependent effects of GTA against *S*. *aureus* biofilm viability. Maximal biofilm viability inhibition (87 and 83%, respectively; *p* < 0.01) was evident with GTA exposures at 2 and 1% concentration. Followed by the applied doses of 0.5, 0.25, and 0.1% concentration, the cell viabilities were observed to be 20, 23, and 35%, respectively (*p* < 0.01). However, the *S*. *aureus* biofilm showed more resistance to 0.05% GTA solution resulting in an only 35% microbial inhibition (*p* < 0.05). Similarly, NIR light was tested to investigate the dose threshold (Fig 2B). A 34% decrease in the biofilm viability was recorded for the light dose of 450 J/cm^2^ (*p* < 0.05), followed by 19 and 7% for 270 and 90 J/cm^2^, respectively.

In Fig 2C, each sample group was irradiated under the fluence of 24, 48, 72, and 96 J/cm^2^, respectively. Above all, the results demonstrated a gradual reduction of the biofilm viability. Under a fluence of 96 J/cm^2^, the biofilm viability showed a significant decrease (47%) whereas a slight change was observed in the case of 24-J/cm^2^ light exposure (around 8% reduction). However, the bacterial biofilm was more susceptible to 48-J/cm^2^ 405-nm laser fluence (15% reduction), followed by 72-J/cm^2^ dose with the reduction of 39% (*p* < 0.01). Fig 2D elaborates higher doses of 405-nm laser irradiation (96, 288, and 480 J/cm^2^) onto the *S*. *aureus* biofilm. Interestingly, the biofilm viability reduction under 96-J/cm^2^ light dosage was equivalent to those from Fig 2C and 2D (47% vs. 43%). Furthermore, only 35% of bacteria were found to be viable under the extreme 405-nm laser irradiation of 480 J/cm^2^ (*p* < 0.01).

### Effect of combined germicides on biofilm viability

**Fig 3:**
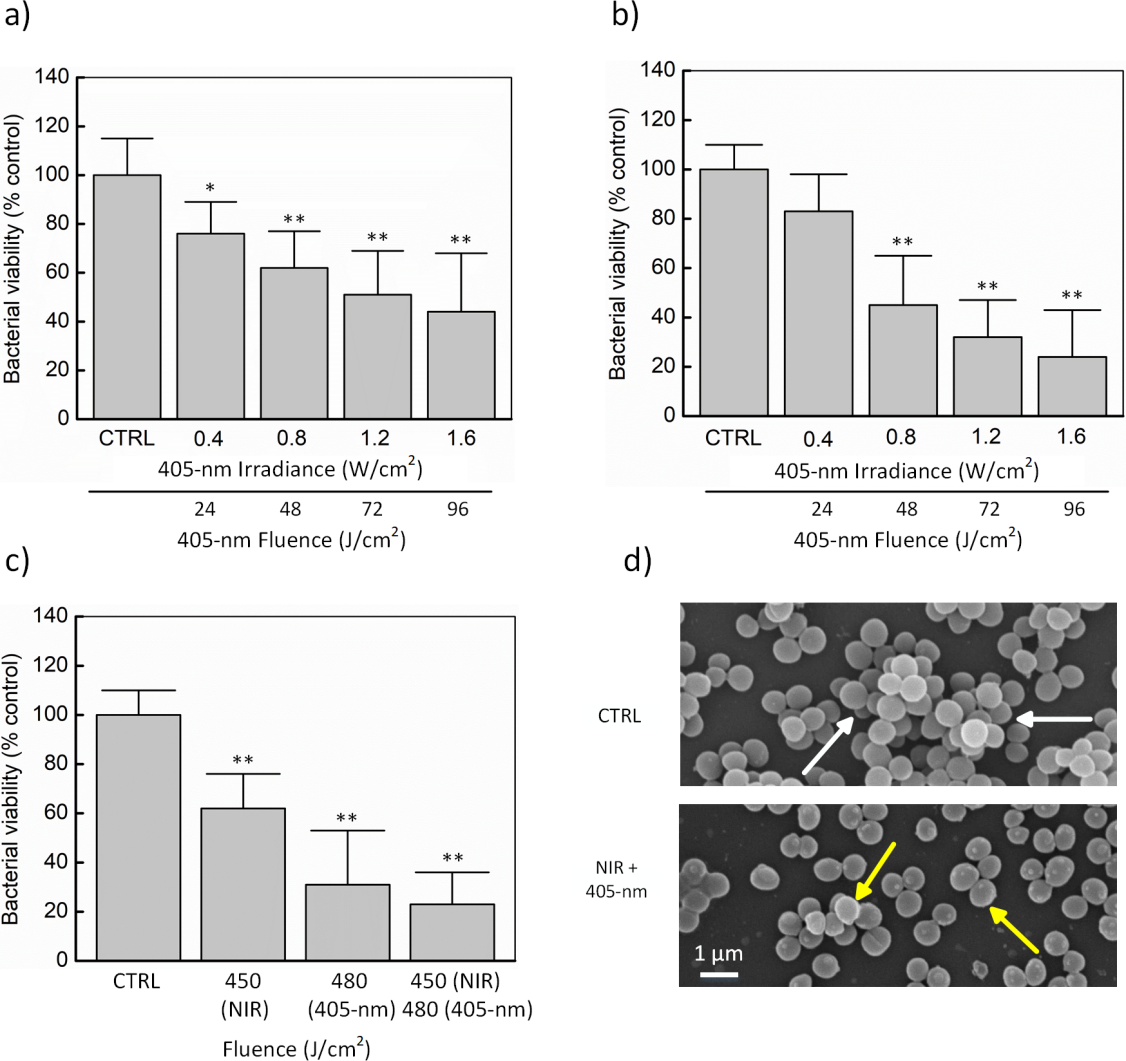
Germicidal combination to decontaminate biofilm on polystyrene surface (24-well plate surface). (a) 60-s NIR light exposure (irradiance = 1.5 W/cm^2^) with various 405-nm laser irradiances for 60 s. (b) 300-s NIR light exposure (irradiance = 1.5 W/cm^2^) with various 405-nm laser irradiances for 60 s. (c) 300-s NIR light (irradiance = 1.5 W/cm^2^) and 300-s 405-nm laser (irradiance = 1.6 W/cm^2^) exposure. (d) SEM images for 300-s NIR light (fluence = 450 J/cm^2^) and 300-s 405-nm laser (fluence = 480 J/cm^2^) exposure. The biofilm viability was estimated by indirectly measuring the metabolic activity of the biofilms with MTT assay (*p < 0.05 and **p < 0.01 vs. untreated control (CTRL); N = 5).

Fig 3 demonstrates the combined effects of different decontaminating factors on *S*. *aureus* biofilm. In Fig 3A, the NIR dose was fixed at 90 J/cm^2^ while 405-nm laser fluence was increased from 24 J/cm^2^ to 96 J/cm^2^. Overall, 90-J/cm^2^ NIR light and 24-J/cm^2^ 405-nm laser exposure could induce a 24% reduction of the biofilm viability whereas the same NIR light fluence in conjunction with 96-J/cm^2^ 405-nm laser application resulted in a 56% reduction in the biofilm viability (*p* < 0.01). The moderate effects could be observed in the cases of 48-J/cm^2^ and 72-J/cm^2^ 405-nm laser modes (38% and 49% reduction, respectively). The similar light dose of 405-nm laser in Fig 3A was also applied in Fig 3B; however, NIR light fluence was raised up to 450 J/cm^2^ (irradiance = 1.5 W/cm^2^ and irradiation time = 300 seconds). For the condition of 450 J/cm^2^ NIR light and 24-J/cm^2^ 405-nm laser activation, a slight reduction of the biofilm viability (just 17%) was observed. By contrast, with increasing 405-nm laser irradiances (0.8, 1.2, and 1.6 W/cm^2^), the biofilm was apparently more susceptible to the light application (55, 68, and 76% reduction, respectively; *p* < 0.01).

This tendency was confirmed by the treatment groups in Fig 3C (NIR light = 450 J/cm^2^ and 405-nm laser = 480 J/cm^2^). Alone NIR light (or alone 405-nm laser) irradiation resulted in a 38% (or 69%) decrease (*p* < 0.01); however, their combination showed a 77% reduction in bacterial viability under the light exposure (*p* < 0.01). Fig 3D gives more details about the biofilm architecture before and after the treatment. The multilayers of the cells in the control samples (white arrows) were destructed into thinner layers (yellow arrows), and the huge difference was observed in the number of cells on the polystyrene surface after 450-J/cm^2^ NIR and 480-J/cm^2^ 405-nm laser exposure.

**Fig 4:**
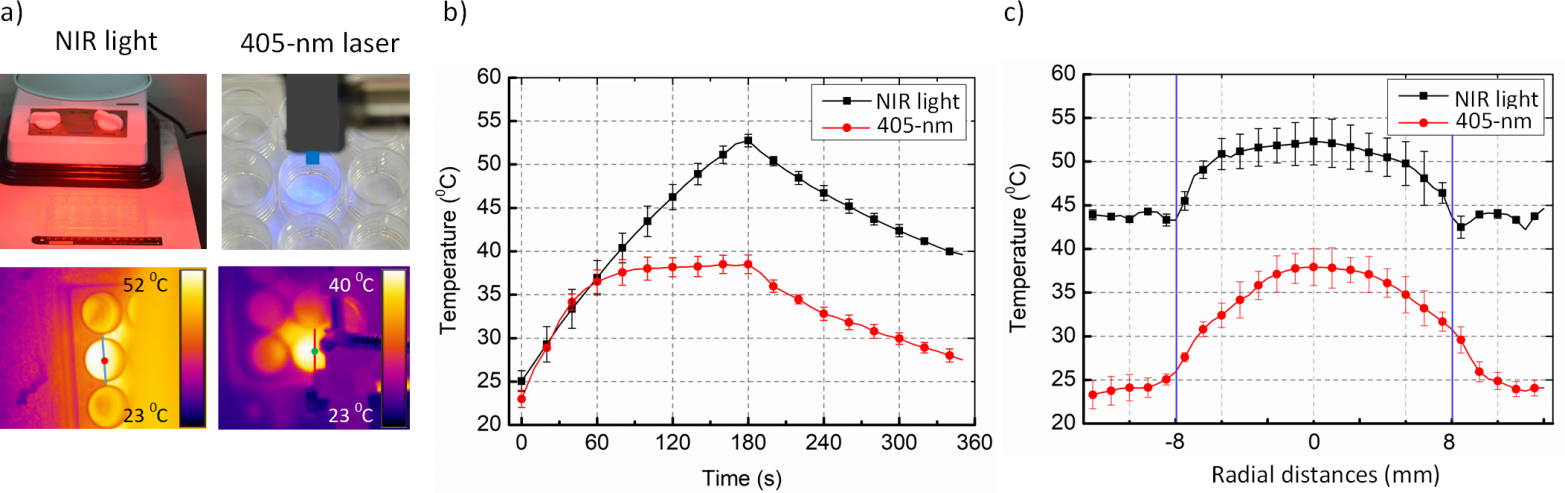
Temperature development during NIR light irradiation (270 J/cm^2^) and 405-nm laser exposure (288 J/cm^2^). (a) Digital (top) and thermal (bottom) images (left: NIR light and right: 405-nm laser). (b) Temperature at center of biofilm surface. (c) Radial temperature on biofilm surface. Red and black color lines represent 405-nm laser and NIR light, respectively (N = 5).

Fig 4 displays the temporal temperature development under NIR light (270 J/cm^2^) and 405-nm laser (288 J/cm^2^) exposure. Digital and thermal images are represented in Fig 4A while Fig 4B and 4C reflect the temperature at the center of the biofilm surface and the radial temperature on *S*. *aureus* biofilm surface, respectively. Overall, the biofilm on the 24-well plate was uniformly irradiated by the NIR light, which also showed a good agreement with the spatial light distribution in S1A and S1B Fig. On the other hand, 405-nm laser was divergently projected on the single well and slightly affected adjacent ones. Although the treatment was intended to deliver for 180 seconds, the recording time was 360 seconds to observe the temperature changes after the light source was turned off.

In Fig 4B, the temperature of the point of interest significantly increased from 23°C to 52°C at the end of NIR light exposure and exponentially decreased to the room temperature. Similarly, under the 405-nm laser irradiation, the temperature at the center of the biofilm surface dramatically rose in the initial 60-s duration and then slightly increased to 38.2°C before exponentially decreasing to 27°C. Fig 4C demonstrates the radial temperature development that follows the light distribution from both NIR and 405-nm laser sources (S1 Fig). The entire surface temperature of biofilm well (radius ~ 8 mm) was almost equivalent to a standard deviation of 8°C for the NIR case while the 405-nm laser irradiation induced a Gaussian-like temperature distribution with the peak temperature of 38.2°C.

**Fig 5:**
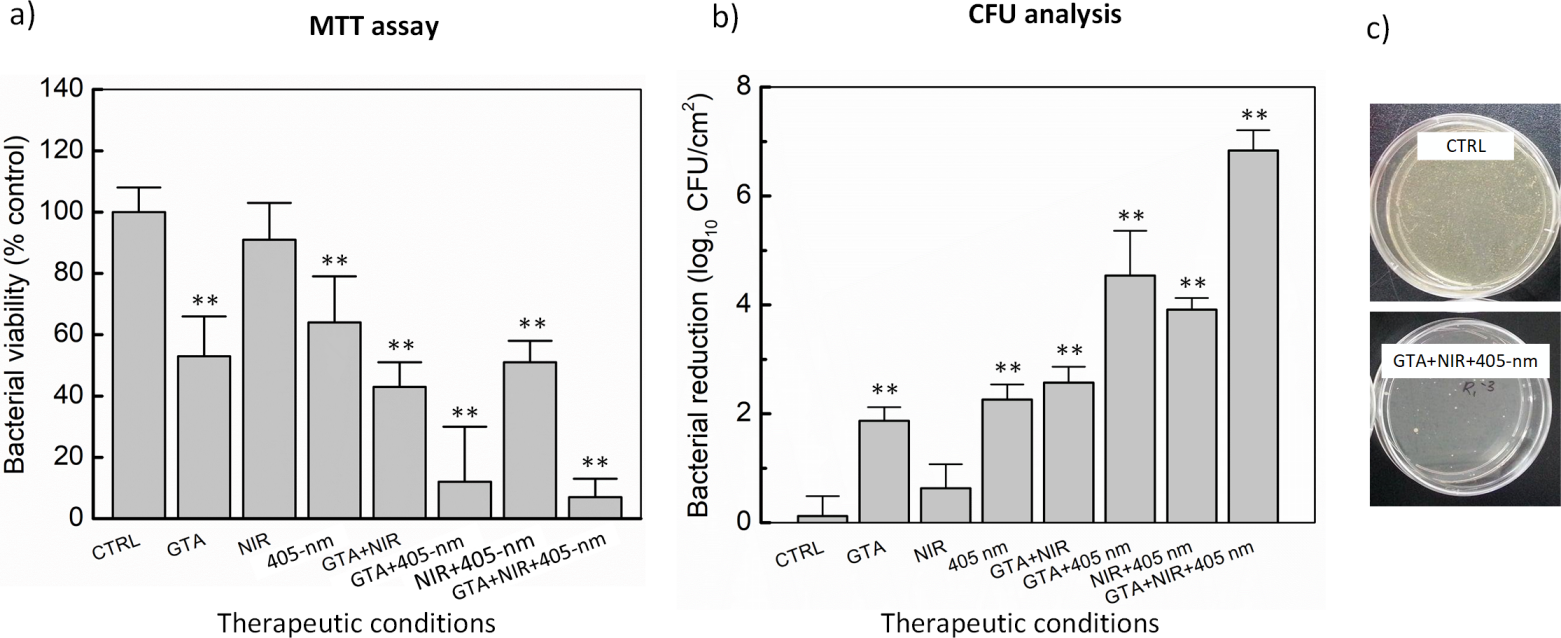
Biofilm inhibitory effect of GTA, NIR light, and/or 405-nm laser exposure on mature *S*. *aureus* biofilm. (a) Measurement of metabolically active cells by MTT assay. (b) Determination of bacterial reduction by CFU analysis. (c) Surviving bacterial colonies on agar plates before and after combined treatment (**p < 0.01 vs. untreated control (CTRL); N = 5).

Fig 5 presents quantitative information on the biofilm inhibitory effect of GTA (0.1%), NIR light (270 J/cm^2^), and 405-nm laser (288 J/cm^2^) exposure on *S*. *aureus* biofilm. Fig 5A and 5B show biofilm quantification determined by MTT assay and CFU analysis while Fig 5C visualizes CFU recovered from mature biofilms before and after the combined treatment. Overall, both the methods well agreed that *S*. *aureus* biofilm was highly susceptible to the combination of GTA, NIR light, and 405-nm laser with the aforementioned doses (7% cell viability and 6.8-log CFU/cm^2^ bacterial reduction for MTT assay and CFU analysis, respectively). In Fig 5A, 91% of biofilm was resistant to the NIR light irradiation, which was 27% and 38% higher than that in the case of either alone 405-nm laser or alone GTA exposure, respectively. Interestingly, the biofilm seemed more susceptible to the combination of GTA and 405-nm laser than either GTA and NIR light or NIR light and 405-nm laser (87, 57, and 49% reduction, respectively; *p* < 0.01). Furthermore, the results of MTT assay were confirmed by determining the viable cells counting method (Fig 5B). Alone 405-nm laser and GTA induced a 2.3 and 1.9-log CFU/cm^2^ reduction, respectively, while NIR light induced a slight inhibition in the bacterial viability (a 0.6-log CFU/cm^2^ reduction). However, the combination of GTA + NIR, NIR + 405-nm laser, and GTA + 405-nm laser reduced the bacterial viability by 2.6, 3.9, and 4.5-log CFU/cm^2^, respectively. Interestingly, the triple combination of GTA, NIR, and 405-nm laser at the optimal dose resulted in 6.8-log CFU/cm^2^ reduction in the viable counts confirming the disinfection action. Fig 5C shows agar plates with the surviving colonies recovered from mature biofilms post-treatment (GTA, NIR light, and 405-nm laser) compared to the untreated control (CTRL). It is noted that the number of CFUs on the control plate directly demonstrated a massive decrease in the treated sample.

### Membrane integrity loss

**Fig 6:**
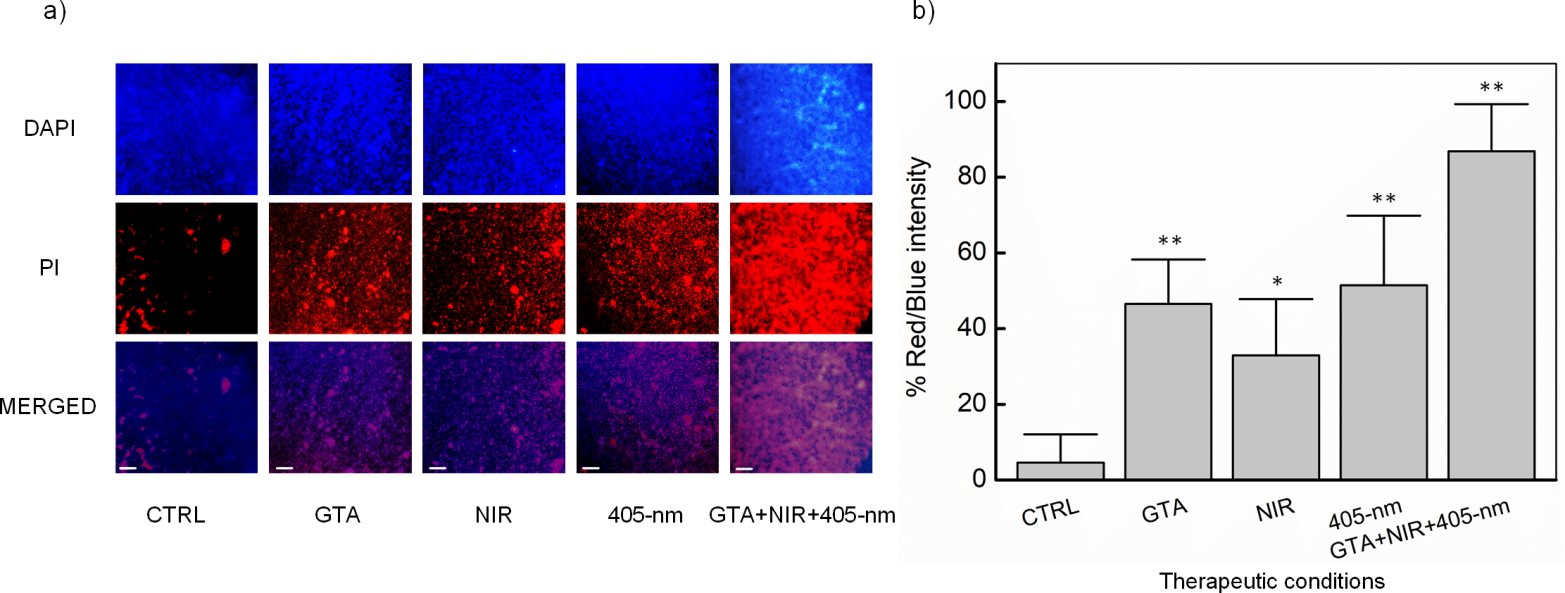
*In situ* visualization of mature *S*. *aureus* biofilms formed on polystyrene surface under various treatment conditions. Dual staining including DAPI (blue) and PI (red) was used to differentiate live and dead cells (*p < 0.05 and **p < 0.01 vs. untreated control (CTRL); N = 5; Scale bar = 30 μm).

The effect of germicide agents on the membrane integrity and permeability was determined by using DAPI and PI dual-stain. PI is a nucleic acid dye, which only binds DNA when membrane integrity is compromised. The confocal laser scanning microscopic (CLSM) images for the effect of individual or combination germicides on biofilm membrane integrity were represented in the Fig 6A and 6B. From the results, it is evident that in the control group, the cells appeared largely blue indicating the intact cell membrane. However, compared with controls, the individual agents exhibited a significantly higher reduction in the membrane permeability. Among the individual germicide agents, GTA (0.1%) and 405-nm laser (288 J/cm^2^) treatment exhibited the higher PI uptake causing 46.5 and 51% loss of membrane permeability, respectively (*p <* 0.01), followed by NIR exposure (32.9%; *p* < 0.05). However, in the case of the combination treatment, the fluorescent intensity of the cells drastically increased, in comparison with the control group as well as the individual germicide groups. The optimal combination of GTA, NIR light, and 405-nm laser caused a significant red/blue intensity (87%, *p* < 0.01), compared with the untreated controls. From these results, it is evident that the combination of GTA, NIR light, and 405-nm laser induced significant membrane permeability.

### Loss of biofilm architecture

**Fig 7:**
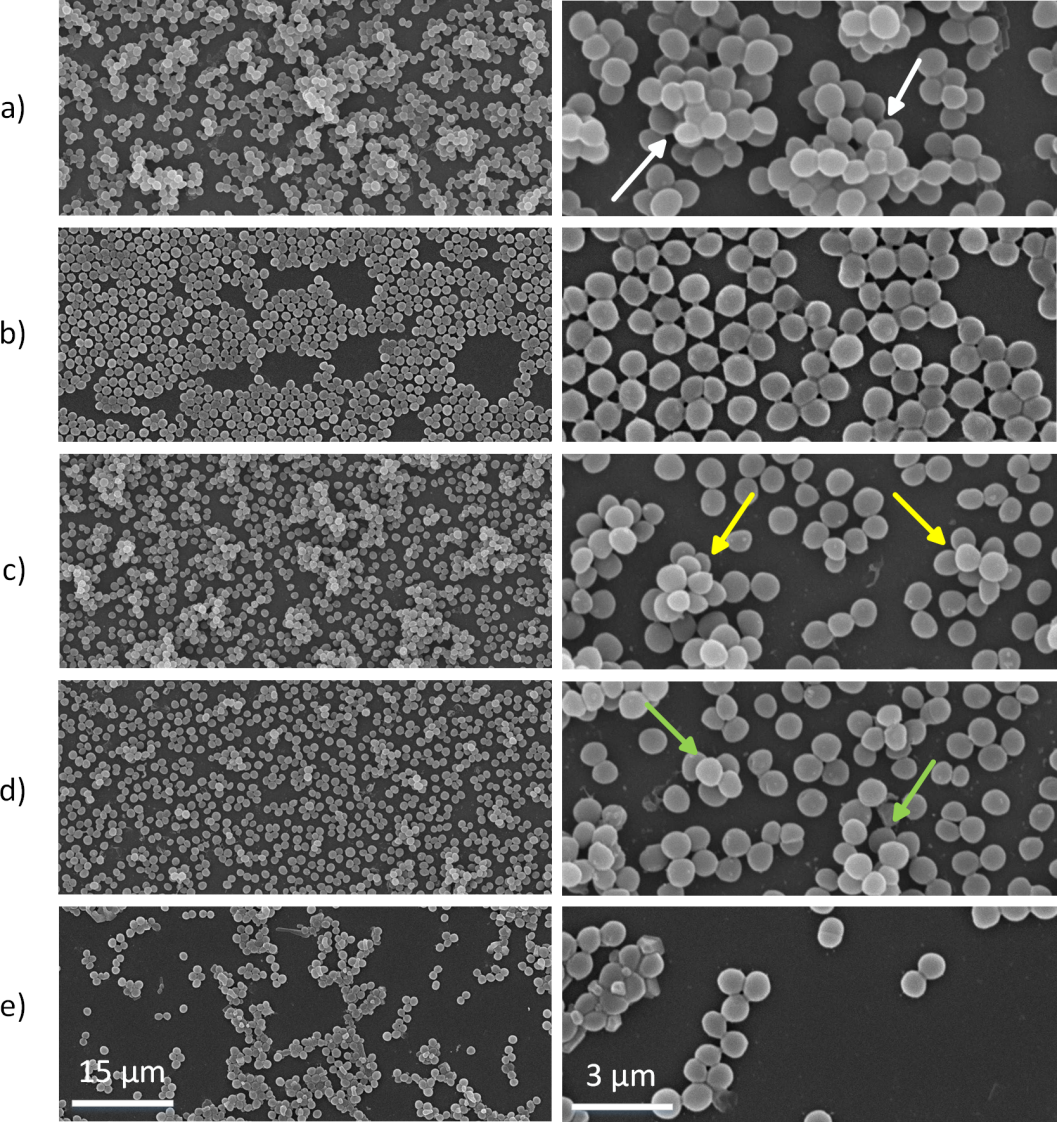
*In situ* architecture visualization of mature *S*. *aureus* biofilm under various treatment conditions. (a) Untreated control. (b) GTA (0.1%). (c) NIR light (270 J/cm^2^). (d) 405-nm laser (288 J/cm^2^). (e) Combination of GTA, NIR light, and 405-nm laser.

As cells were found increasingly susceptible to the individual germicides at the optimal concentrations, SEM experiment was performed in order to learn the effects of different individual agents on biofilm morphology. From Fig 7, it is evident that control biofilms exhibited limited exopolymeric matrix of the cells which were arranged in multi-layers in three-dimensional spaces and attached to each other as indicated by white arrows.

On the other hand, single treatments (GTA, NIR, and 405-nm laser) exhibited a moderate damage to the biofilm architecture compared to the control. In other words, these conditions exhibited altered biofilm appearance with the scattered *S*. *aureus* cells and a reduced exopolymeric matrix surrounding the cells (yellow arrows in Fig 7C and green arrows in Fig 7D). Furthermore, the combined treatment (GTA, NIR, and 405-nm laser) significantly reduced the cell viability of the biofilm as many cells appeared reduced in size, lacked cell membrane integrity and permeability.

## Discussion

Healthcare-associated infections (HAIs) have increasingly become problematic in the endoscopic procedures resulting in several severe diseases such as CRE-related infections, pneumonia, and bacteremia. However, many previous studies have agreed that some bacterial strains are currently resistant to traditional antimicrobials [10, 11]. They have adopted various mechanisms to improve their resistance towards disinfectants. Therefore, any promising approaches should be attempted to treat bacterial infections, especially those arose from drug-resistant bacteria. Some combination methods have been introduced to the literature including light-based or coating-based solutions.

In this study, three germicides (GTA, NIR light, and 405-nm laser) were used either alone or in combinations against the mature biofilms of *S*. *aureus* on the polystyrene surface. To investigate the dose for each agent in the combined state, the rapid metabolic activity determination of each agent was carried out using MTT assay. Previous reports suggested that MTT assay was useful in the rapid evaluation of bacterial viability and in presence of various physical and chemical agents to bypass the labor-intensive and time-consuming steps of counting CFU or disposing of radio-isotopic materials [45–48]. As showed in Fig 2A, the mature biofilms of *S*. *aureus* used in the present study were susceptible to GTA treatment in the concentration-dependent manner (i.e., around 65% bacterial inhibition under 0.1% GTA exposure in 180 s). The effectiveness of alone NIR against biofilm viability was then investigated, which showed that NIR exposure at 270 J/cm^2^ caused around 19% biofilm inhibition (Fig 2D). These results are consistent with the earlier observations that NIR light was not effective when used alone against *S*. *aureus* [49]. Further, 405-nm laser at 288 J/cm^2^ resulted in about 58% viability loss in *S*. *aureus* biofilms (Fig 2D) and these results are consistent with the earlier reports on 405-nm light which exhibited 62% reduction in the *S*. *aureus* biofilms [50]. It was also noted from the Fig 2B and 2D that the light dose between NIR light and 405-nm laser (270 J/cm^2^ vs. 288 J/cm^2^ as an example) were comparable. Additionally, the combined effect of the anti-biofilm activity of NIR light and 405-nm laser irradiation was investigated by modulating fluence and exposure time (Fig 3). When the biofilms were exposed to the combination of NIR light (450 J/cm^2^) and 405-nm laser (480 J/cm^2^) for 300 s, there was a significant reduction (around 77 ± 13%) in the biofilm viability. Interestingly, these results are comparable to the bactericidal effect of NIR-UV (ultraviolet) combination (around 99.9%) against gram-negative pathogens [51, 52].

Fig 4 depicts photo-thermal effects in both cases of NIR light and 405-nm laser exposure. Although NIR light was irradiated in a wide region (40-mm radius), only plate wells in the presence of bacteria mainly absorbed the light and converted into the heat (Fig 4A; lower-left corner). As the reflection of the uniform distribution of NIR light in S1 Fig, the radial temperature development on the bacterial surface exponentially decreased towards the boundary with a slight difference of 8°C. By contrast, the temperature distribution for 405-nm laser case was the more Gaussian shape due to the truly Gaussian light source. Therefore, the bactericidal effect in the center of the well may be higher than that in the boundary of the well. The maximum temperature of 38.2°C also evidenced that 405-nm laser inactivated the *S*. *aureus* biofilm due to photoexcitation mechanisms, unrelated to any indirect heating effects.

One best approach to control the biofilm is through the combinational use of multiple disinfectants with diverse mechanisms of action. Based on the preliminary results, GTA (0.1%), NIR light (270 J/cm^2^), and 405-nm laser (288 J/cm^2^) were chosen as the promising combination. This combination resulted in significantly inhibited biofilm viability by 93% (MTT method) and 6.8-log CFU/cm^2^ (CFU analysis) which is comparable with the disinfection nature earlier reported [53, 54]. The disinfection mechanism of the combined lethal effect under GTA, NIR light, and 405-nm laser exposure on *S*. *aureus* biofilms was qualitatively determined by CLSM images (Fig 6). The triple combination (GTA, NIR light, and 405-nm laser) resulted in biofilms cells with more PI fluorescence compared to the controls. In addition, from SEM images (Fig 7), the triple combination significantly damaged the biofilm architecture and cell envelop of the test bacteria compared to control bacteria. These results are consistent with log CFU reductions in the viable counts (Fig 5B) and quantitative results of the membrane damage (PI uptake) assay (Fig 6B). From the results, it was noted that the cell membrane damage was the main factor responsible for the disinfection of GTA, NIR light, and 405-nm laser combination. Sequential exposure of GTA and NIR light may lead to biofilm dehydration and membrane integrity loss which eventually facilitates 405-nm laser to penetrate the biofilm cells and generate free radicals based on oxygen-dependent photoexcitation mechanism to cause membrane damage and cell viability loss. Specifically, aldehyde radicals in GTA formed protein-protein crosslinks due to the alkylation of hydroxyl (-OH), carbonyl (C = O) and amino (-NH_2_) groups [55, 56]. GTA reacts with the amino group of a biofilm protein to form a methylene bridge, which is then linked with another protein chain in the cells. Therefore, water may be eventually removed from the biofilm (dehydration effect) and resulting in the inhibition of protein, DNA, and RNA synthesis. As a consequence, the remaining water on the biofilm surface absorbed the NIR light to convert into the heat to destroy bacteria via various thermal effects such denaturation of the cell wall and protein/enzymes, leading to the leakage of cellular content and bacterial death [29]. Finally, 405-nm laser exposure induced an oxygen-dependent photoexcitation reaction within the exposed microbes [12]. Stimulated endogenous intracellular porphyrins then absorbed the photons and brought to the massive production of the cytotoxic reactive oxygen species (ROS) such as -OH, H_2_O_2_, and especially singlet oxygen O_2_, which was considered as a trigger of cell injury [12, 57]. Once ROS was intracellularly accumulated, it could cause the widespread injury to important cellular structure, leading to the disruption of the cytoplasmic content and cell walls of the exposed *S*. *aureus* bacteria. This result was in a good agreement with the study of Murdoch *et al*. (2012) [43] that bacteria were more susceptible in the drying surfaces such as polyvinyl chloride (PVC) and acrylic materials than in the liquid suspensions.

Although the current study demonstrated the promising efficacy of GTA, NIR light, and 405-nm laser combination, some limitations should be addressed to fill in the gap of the results. Firstly, the NIR light and 405-nm laser should be uniformly collimated to avoid any unexpected side-effects over the biofilm surface. Based upon the collimated beam, one could better focus the light on the target treatment area, not to affect the adjacent wells. Moreover, all the results in the current study were acquired as the final effects of antimicrobial activity through MTT assay, CFU analysis, fluorescent, and SEM images. Therefore, more advanced quantitative methods such flow cytometry should be employed for the detection of ROS production during the treatment [10, 11, 40] to further validate the current findings. The exact function of endogenous intracellular porphyrins in the disinfection process resulting from 405-nm exposure should be fully elucidated. In addition, only *S*. *aureus* strain was evaluated in the current study. Thus, other strains and bacteria should be tested to assess the different susceptibility to three aforementioned agents as gram-negative bacteria are usually less susceptible to antibiotics due to the presence of the outer membrane [32].

The preliminary results in the current study were promising to translate into in vivo testing and even clinical applications such as disinfecting the bacterial biofilm in the endoscope. Therefore, the future study will test Teflon tubing models, which can mimic the bacterial biofilm forming inside the endoscope channels. The NIR light will be altered by the diode laser system (monochromatic wavelength = 808 nm) and joined with the current 405-nm laser via the fiber coupler. Eventually, the energy from the two laser beams will be simultaneously delivered through a customized basket-integrated optical diffusing applicator to uniformly and circumferentially irradiate the biofilm inside the tubes and to inactivate the bacterial biofilm.

## Conclusions

The current study investigated the combined effects of GTA, NIR light, and 405-nm laser on *S*. *aureus* biofilm inactivation. The combined method could induce around 6.8-log reduction of bacterial biofilm. Therefore, the proposed technique may be a feasible solution for endoscope reprocessing to minimize any secondary infection to other patients.

## Supporting information

S1 Fig **Spatial light emission measurement in 2D (a and c) and 3D (b and d).** (a) and (b) NIR light. (c) and (d) 405-nm laser (*I*_*0*_: maximum light intensity and *σ*: beam radius of 405-nm laser determined at 13.5%×*I*_*0*_).
(TIF)

S2 Fig *In situ* architecture visualization of *S*. *aureus* biofilm under combined exposures. (a) GTA (0.1%) + NIR light (270 J/cm^2^). (b) GTA (0.1%) + 405-nm laser (288 J/cm^2^). (c) NIR light (270 J/cm^2^) + 405-nm laser (288 J/cm^2^) (N = 3).
(TIF)

S1 Table Light doses for preliminary tests in four individual groups (N = 5). (DOC)

S2 Table Light doses for preliminary tests in three combined groups (N = 5). (DOC)
