# Supplementary figures and images for: Antibacterial activity of Staphylococcus aureus biofilm under combined exposure of glutaraldehyde, near-infrared light, and 405-nm laser
Source: PLoS One. 2018 Aug 27;13(8):e0202821. doi: 10.1371/journal.pone.0202821 (PMC6110465; doi:10.1371/journal.pone.0202821)

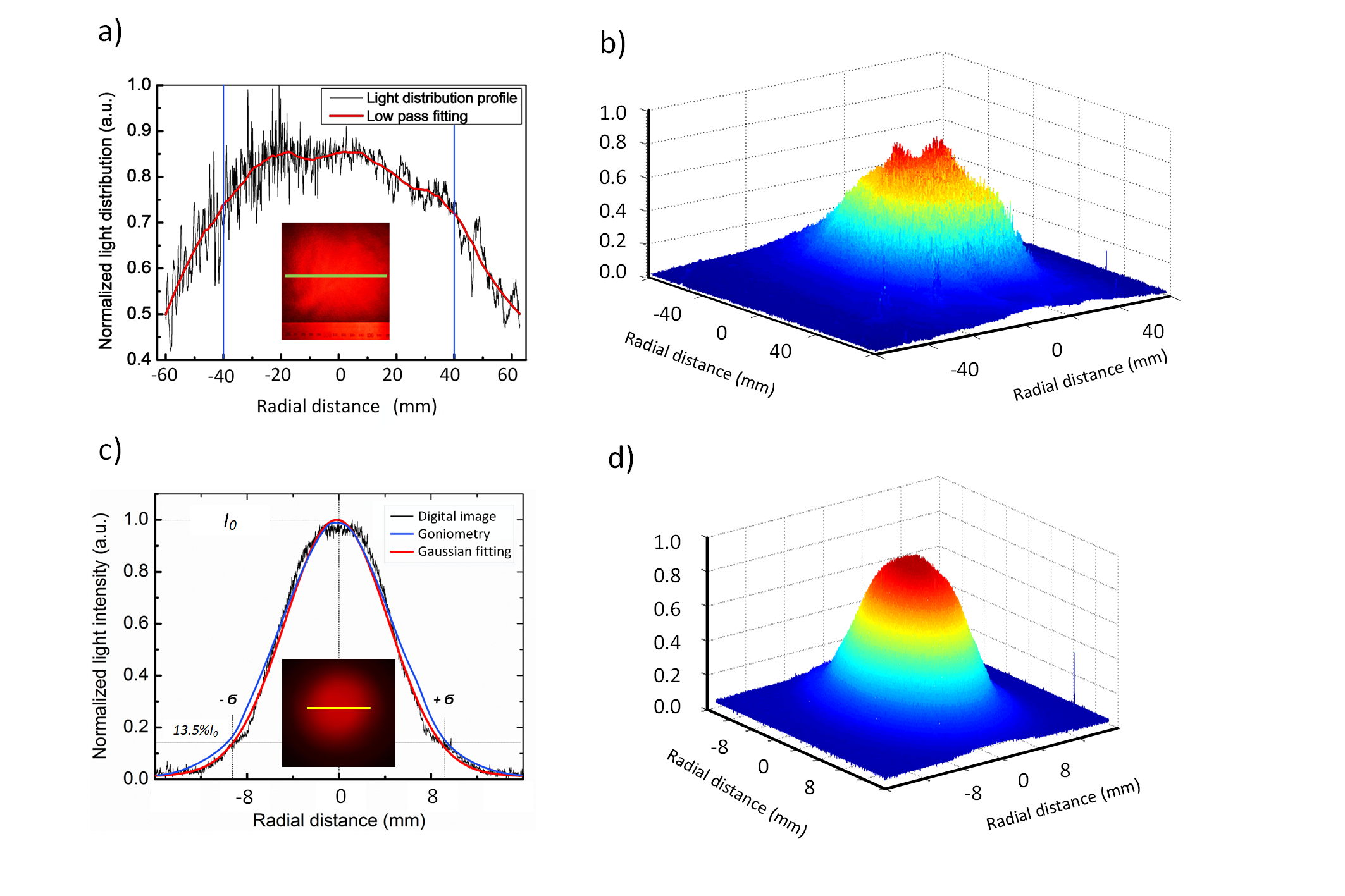

Supplement: S1 Fig — Spatial light emission measurement in 2D (a and c) and 3D (b and d). (a) and (b) NIR light. (c) and (d) 405-nm laser (I0: maximum light intensity and σ: beam radius of 405-nm laser determined at 13.5%×I0). (TIF) [file pone.0202821.s001.tif]

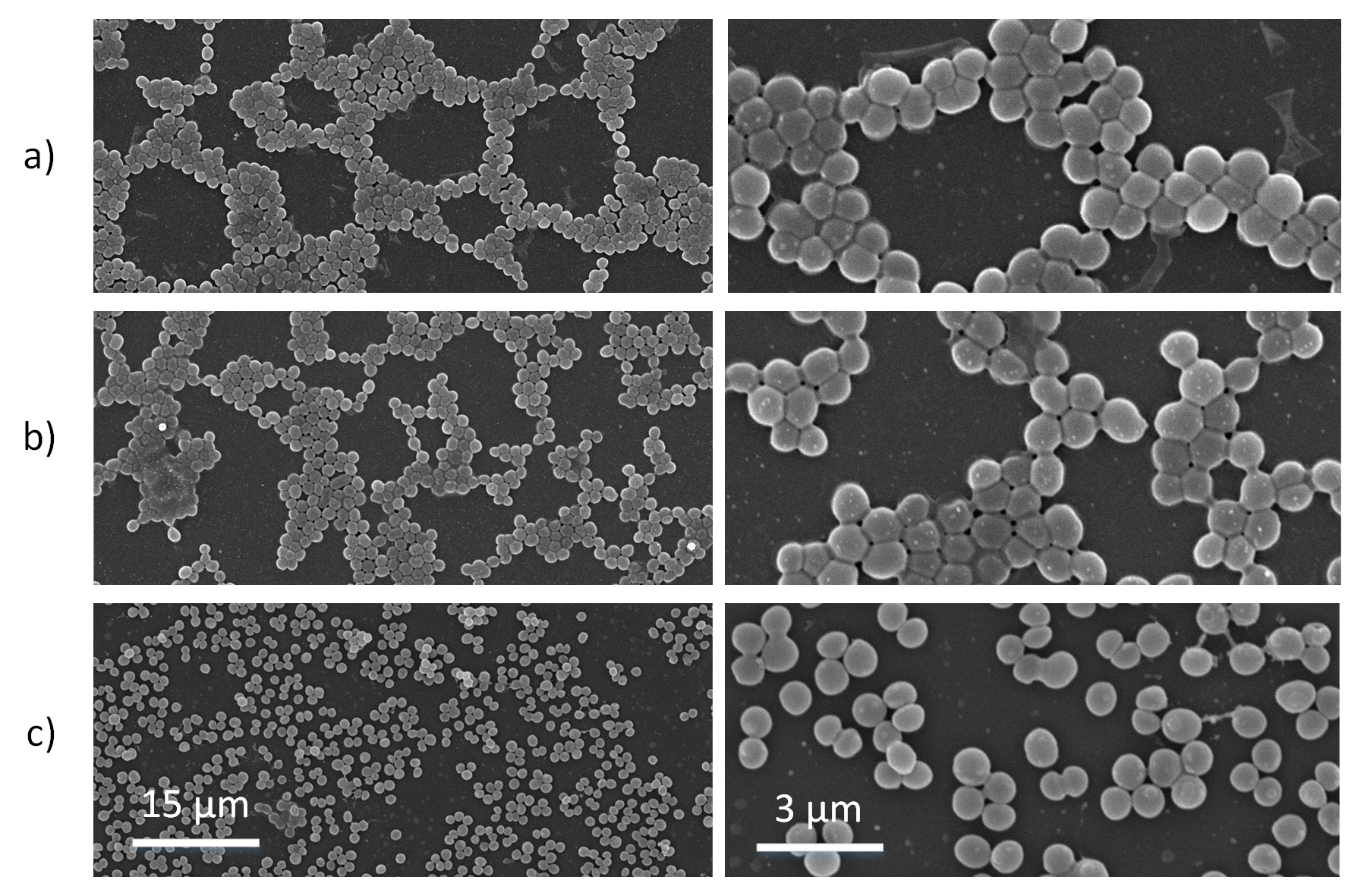

Supplement: S2 Fig — (a) GTA (0.1%) + NIR light (270 J/cm2). (b) GTA (0.1%) + 405-nm laser (288 J/cm2). (c) NIR light (270 J/cm2) + 405-nm laser (288 J/cm2) (N = 3). (TIF) [file pone.0202821.s002.tif]
