# Supplementary material for: Antibacterial activity of Staphylococcus aureus biofilm under combined exposure of glutaraldehyde, near-infrared light, and 405-nm laser
Source: PLoS One. 2018 Aug 27;13(8):e0202821. doi: 10.1371/journal.pone.0202821 (PMC6110465; doi:10.1371/journal.pone.0202821)
Supplement: S1 Table — (DOC) [file pone.0202821.s003.doc]

**S1 Table.**

| **Test** | **Germicidal exposure** | **Irradiance (W/cm^2^)** | **Exposure Time (s)** | **Fluence**  **(J/cm^2^)** | **Bacterial viability (%)** | **Standard deviation (%)** |
| --- | --- | --- | --- | --- | --- | --- |
| Test 1 | GTA | Concentration (%) = 0.05, 0.1, 0.25, 0.5, 1, 2; Bacterial viability (%) = 65 ± 10, 35 ± 4, 23 ± 2, 20 ± 2, 17 ± 3, and 13 ± 6, respectively. | | | | |
|  | | | | | | |
| Test 2 | NIR light | 1.5 | 60 | 90 | 93 | 12 |
|  |  |  | 180 | 270 | 81 | 25 |
|  |  |  | 300 | 450 | 66 | 27 |
|  | | | | | |  |
| Test 3 | 405-nm laser | 0.4 | 60 | 24 | 92 | 21 |
|  |  | 0.8 |  | 48 | 85 | 23 |
|  |  | 1.2 |  | 72 | 61 | 15 |
|  |  | 1.6 |  | 96 | 53 | 32 |
|  | | | | | |  |
| Test 4 | 405-nm laser | 1.6 | 60 | 96 | 57 | 35 |
|  |  |  | 180 | 288 | 42 | 22 |
|  |  |  | 300 | 480 | 35 | 27 |

** Fluence (J/cm^2^) = irradiance (W/cm^2^) × exposure time (s)*
