# Supplementary material for: Antibacterial activity of Staphylococcus aureus biofilm under combined exposure of glutaraldehyde, near-infrared light, and 405-nm laser
Source: PLoS One. 2018 Aug 27;13(8):e0202821. doi: 10.1371/journal.pone.0202821 (PMC6110465; doi:10.1371/journal.pone.0202821)
Supplement: S2 Table — (DOC) [file pone.0202821.s004.doc]

**S2 Table.**

| **Test** | **Germicidal exposure** | **Irradiance (W/cm^2^)** | **Exposure Time (s)** | **Fluence**  **(J/cm^2^)** | **Bacterial viability (%)** | **Standard deviation (%)** |
| --- | --- | --- | --- | --- | --- | --- |
| Test 5  IR light  (fluence = 90 J/cm^2^) | 405-nm laser | 0.4 | 60 | 24 | 76 | 13 |
|  |  | 0.8 | 60 | 48 | 62 | 15 |
|  |  | 1.2 | 60 | 72 | 51 | 18 |
|  |  | 1.6 | 60 | 96 | 44 | 24 |
|  | | | | | |  |
| Test 6  IR light  (fluence = 450 J/cm^2^) | 405-nm laser | 0.4 | 60 | 24 | 83 | 15 |
|  |  | 0.8 | 60 | 48 | 45 | 20 |
|  |  | 1.2 | 60 | 72 | 32 | 16 |
|  |  | 1.6 | 60 | 96 | 24 | 17 |
|  | | | | | |  |
| Test 7 | NIR light | 1.5 | 300 | 450 | 62 | 14 |
|  | 405-nm laser | 1.6 | 300 | 480 | 31 | 22 |
|  | NIR light | 1.5 | 300 | 450 | 23 | 13 |
|  | 405-nm laser | 1.6 | 300 | 480 |  |  |

** Fluence (J/cm^2^) = irradiance (W/cm^2^) × exposure time (s)*
